# *Aspergillus* serology for chronic pulmonary aspergillosis diagnosis: optimization of an enzyme-linked immunosorbent assay kit and assessment of a Western blot kit performance

**DOI:** 10.1128/jcm.00182-26

**Published:** 2026-05-28

**Authors:** Jeanne Bigot, Charles Gibert, Nicolas Millet, Wissem Abderrahim, Sophie Thorez, Yaye Senghor, Jacques Cadranel, Cendrine Godet, Christophe Hennequin, Thomas Maitre, Juliette Guitard

**Affiliations:** 1 Sorbonne Université, Inserm, Centre de Recherche Saint-Antoine, CRSA, AP-HP, Hôpital Saint-Antoine, Service de Parasitologie-Mycologie, Paris, France; 2 GRC40 SoLID, SOrbonne study group for Lung Infectious Diseases, Sorbonne Université 27063, Paris, France; 3 Service de Parasitologie-Mycologie, Assistance Publique-Hôpitaux de Paris (AP-HP), Hôpital Saint-Antoine et Sorbonne Université, Paris, France; 4 Sorbonne Université, Inserm, Centre de Recherche Saint-Antoine, CRSA 551865, Paris, France; 5 Service de Pneumologie et d’Oncologie Thoracique, Assistance Publique-Hôpitaux de Paris (AP-HP), Hôpital Tenon et Sorbonne Université, Paris, France; 6 Sorbonne Université, Centre d'Immunologie et des Maladies Infectieuses (Cimi-Paris), Paris, France; University of Utah, Salt Lake City, Utah, USA

**Keywords:** chronic pulmonary aspergillosis, serology, diagnostic, ELISA, Western blot

## Abstract

Chronic pulmonary aspergillosis (CPA) is a progressive and potentially fatal lung disease occurring in patients with underlying structural lung damage. Its diagnosis remains challenging because clinical and radiological features are often nonspecific and overlap with pre-existing respiratory conditions. Detection of anti-*Aspergillus* IgG antibodies is a cornerstone of diagnosis, as microscopy and culture have limited sensitivity. Several serological assays are available, including enzyme-linked immunosorbent assay (ELISA), which is highly sensitive but may lack specificity, and Western blot (WB), for which diagnostic performance data are limited. This study aimed to optimize the serological strategy for CPA diagnosis. The diagnostic performance of a commercial ELISA assay for *Aspergillus*-specific IgG (Platelia *Aspergillus* IgG, Bio-Rad) was optimized using serum samples collected at diagnosis from CPA patients and control subjects. Receiver operating characteristic (ROC) curve analysis was performed to assess alternative cut-off values. In parallel, the performance of a commercial WB assay (LDBio) was retrospectively evaluated using serum samples from five groups: CPA, *Aspergillus* colonization, airway contamination, negative controls with chronic respiratory disease, and healthy donors. ROC curve analysis showed that increasing the ELISA cut-off from 10 to 25 AU/mL maintained excellent sensitivity while improving specificity from 83.5% to 92.8%. The WB assay demonstrated a sensitivity of 87.0% for CPA diagnosis; however, specificity varied depending on the control group, ranging from 41.4% to 60.7%. Adjusting the ELISA threshold to 25 AU/mL significantly enhances diagnostic specificity without compromising sensitivity, whereas WB provides limited additional diagnostic value due to suboptimal specificity.

**IMPORTANCE:**

Chronic pulmonary aspergillosis (CPA) is a severe and often overlooked fungal lung disease that occurs in patients with underlying structural pulmonary disorders. Diagnosis is challenging because clinical and radiological features are frequently nonspecific and overlap with pre-existing lung conditions. Detection of anti-*Aspergillus* IgG is central to diagnosis, but the optimal interpretation of available assays remains uncertain. In this study, we assessed the diagnostic performance of serological tests using well-defined patient groups reflecting real-world clinical practice. We show that increasing the cut-off of a widely used ELISA assay significantly improves diagnostic specificity without compromising sensitivity. In contrast, our results suggest that a commercial Western blot test provides only limited diagnostic value due to suboptimal specificity, clearly demonstrated using the different patient groups studied. These results help refine the serological strategy for CPA diagnosis and improve its clinical interpretation.

## INTRODUCTION

Depending mainly on the host’s immune status, *Aspergillus* respiratory infection presents in different clinical forms (1). Whereas invasive aspergillosis occurs in strongly immunocompromised patients, most often unable to mount an effective immune response against *Aspergillus*, chronic pulmonary aspergillosis (CPA) and allergic broncho-pulmonary aspergillosis (ABPA) are characterized by a significant production of anti-*Aspergillus* IgG. CPA occurs in patients with previously damaged lung parenchyma or bronchus due to underlying chronic lung disease, such as chronic obstructive pulmonary disease, emphysema, post-tuberculous cavities, or bronchiectasis (2). The global annual incidence of CPA was estimated to be 1.8 million cases, with 304,000 deaths (3). Despite appropriate therapeutic management, the slowly progressive and destructive parenchymal lung disease, mostly caused by *Aspergillus fumigatus* (4)*,* is responsible for a 5-year mortality rate of up to 45% (2, 5). Clinical symptoms and radiological findings are often nonspecific, and mycological culture of pulmonary samples presents low sensitivity, between 26% and 81%, depending on case series (6, 7). Therefore, in this setting, serology is a key diagnostic tool for CPA (8, 9). Serum anti-*Aspergillus* IgG are detected by different methods, including enzyme immunoassays (EIA), immunoprecipitation-based techniques (allowing the demonstration of precipitin lines), Western blot (WB), and immunochromatographic assays. EIA encompasses different technologies, with the enzyme-linked immunosorbent assay (ELISA) and the fluorescent enzyme immunoassay (FEIA) being the most used (10). Thanks to their automation, EIA tests allow a large number of serum samples to be tested in a limited amount of time. Some of these diagnostic kits also offer the advantage of using recombinant antigens, which, theoretically, should improve reproducibility. Finally, providing concentration standards that must be tested in parallel, some of these kits allow a quantitative determination of anti-*Aspergillus* IgG levels (11). EIA tests are sometimes seen as screening methods, thanks to their excellent sensitivity. Their specificity for diagnosing CPA is much debated, as bronchial colonization can be associated with anti-*Aspergillus* IgG (12). On the other hand, techniques based on immunoprecipitation, which have been considered as the gold standard of *Aspergillus* serology until recently, are labor-intensive and non-standardized. Furthermore, reading precipitin lines is subjective, and the interpretation of results depends on the laboratory, making comparison of results between centers almost impossible (10). A commercial version of WB is available for detecting anti-*Aspergillus* antibodies. The technique remains cumbersome, and the results are reader-dependent and could be potentially antigen-batch-dependent. Immunoprecipitation-based techniques and WB are thought to be more specific, counterbalancing the supposed “over-sensitivity” of EIA techniques (13). In France, a diagnostic strategy is frequently used that begins with a screening method, such as the ELISA test, followed, in the event of positive results, by a more specific test, such as precipitation tests or Western blot (14). While the performance of EIA methods has been extensively studied for the diagnosis of CPA (15–17), data are more scarce about the diagnostic performance of the *Aspergillus* Western blot kit in this setting (18–20).

The primary objective of this study was to optimize our serological strategy for diagnosing chronic pulmonary aspergillosis. We first sought to refine the interpretation of the results obtained from a commercial ELISA assay. Subsequently, we evaluated the performance of the Western blot in distinguishing chronic pulmonary aspergillosis from other *Aspergillus* conditions.

## MATERIALS AND METHODS

### Patient selection, data collection, and definitions

For the first part of the study, which focused on the ELISA technique, a panel of 188 sera was selected from 188 patients. All 36 patients with a definite diagnosis of CPA met the diagnostic criteria for CPA according to the ESCMID/ERS definition (8) (Group 1). CPA was diagnosed based on the combination of clinical symptoms lasting at least 3 months, compatible imaging lesions, and positive serology. Serology was performed using the Bio-Rad ELISA test. Samples with positive (>10 AU/mL) or borderline (>5 AU/mL) results were subsequently analyzed by an in-house immunoelectrophoresis assay. Only sera for which the IEP result confirmed the presence of anti-*Aspergillus* IgG were considered positive for anti-*Aspergillu*s IgG. All these sera were drawn before the initiation of any antifungal treatment or surgery. The control groups (groups 2, 3, 4, and 5) consisted of 152 sera from patients with other pulmonary diseases who were suspected of having *Aspergillus* disease, but in whom a diagnosis of CPA and ABPA was excluded after a 6-month follow-up period. Among these patients, 46 had *Aspergillus* bronchial colonization, defined by repeated positive culture for *Af* in at least two respiratory samples obtained 3 months apart (Group 2), 30 patients were suspected of airway contamination, defined by *Af* positive culture in a single respiratory sample (among several collected three months apart) (Group 3), and 71 patients were suspected of having either CPA or ABPA but whose mycological culture results were negative and a 6-month follow-up excluded the diagnosis of any *Aspergillus* disease (Group 4). Finally, five sera were drawn from healthy donor patients (pregnant women, Group 5). For groups 2, 3, 4, and 5, the values of anti-*Aspergillus* IgG titers measured by ELISA were recorded after patients were classified into the different groups.

Within this panel, a subgroup of 80 patients was randomly selected to assess the performance of the Western blot assay. Thus, Group 1 was reduced to 23 patients, Group 2 to 29 patients, Group 3 to 12 patients, and Group 4 to 11 patients. Group 5 was constituted of the same five patients.

### Serology methods

All serum samples have previously been tested for anti-*Aspergillus* antibody using a commercial ELISA. In the case of positive (>10 AU/mL) or borderline (>5 AU/mL) results, they were submitted to a home-made immunoelectrophoresis. All sera were stored frozen at −20°C before testing.

#### ELISA

Serological analysis by ELISA was performed using the Platelia *Aspergillus* IgG (Bio-Rad, Marnes-la-Coquette, France). The assays were conducted according to the manufacturer’s instructions. The technique was automated using an Evolis automated microplate system (Bio-Rad). A positive result for *A. fumigatus*-specific IgG was defined as a concentration ≥10 AU/mL, in accordance with the manufacturer’s recommendations and prior studies on CPA diagnosis (21), while those between 5 and 10 AU/mL were considered “intermediate.” When the measured titers were >80 AU/mL, serum dilutions were not performed.

#### Aspergillus Western blot

Serological analysis by WB was performed using the *Aspergillus* Western Blot IgG assay (LDBio Diagnostics, Lyon, France). The assays were conducted retrospectively, in accordance with the manufacturer’s instructions. Briefly, a membrane was incubated with the patient’s serum, allowing specific IgG antibodies to bind to the immobilized antigens. After washing, an enzyme-conjugated anti-human IgG antibody was added, and the reaction was visualized using a chromogenic substrate. The resulting band pattern was compared with a reference profile. The simultaneous presence of at least two well-defined bands among P16, P18–20, P22, and P30 indicates the presence of specific anti-*Aspergillus* antibodies; otherwise, the assay was considered negative.

#### Immunoelectrophoresis

Immunoelectrophoresis was performed on ready-to-use agarose gels (Hydragel, Sebia, France) using a mixture (1:1) of somatic and culture filtrate *Aspergillus fumigatus* antigens (FKS1; Microgen Bioproducts, UK) and patient sera. After migration for 30 min at 100 V, gels were incubated in barbital buffer (pH 9.2) for 48 h at room temperature, then washed, dried, stained with amido black, and destained before reading. Positive and negative controls were included in each run. Gels were read independently by two technicians, and sera were considered positive when ≥2 precipitin lines were observed. Sequential sera from the same patient were analyzed in a single run (11).

### Statistical analysis

To optimize the performance of the ELISA method, we performed a receiver operating characteristic (ROC) analysis to identify the cut-off value that maximizes sensitivity and specificity. Area under the curve (AUC), Youden statistic test, likelihood ratios (LR+ and LR−), positive and negative predictive values (PPV and NPV) were also calculated. For predictive values, we used the prevalence reported by Maitre et al. at 0.000308, considering the general population in Paris, France (2). These calculations were done using definite cases of CPA (Group 1) versus control groups (i.e., Groups 2, 3, 4, and 5).

In a second step, we assess the value of WB to distinguish between CPA and control groups. Sensitivity, specificity, LR+, LR−, PPV, and NPV were calculated for the WB technique by comparing results from CPA cases and the different control groups.

The likelihood ratios were calculated to assess the diagnostic performance of the assays. An LR+ >10 was considered to provide strong evidence in favor of the disease, while an LR+ between 5 and 10 was interpreted as moderate evidence. Conversely, an LR− <0.1 was considered to provide strong evidence against the disease, and an LR− between 0.1 and 0.2 was interpreted as moderate evidence against it.

The agreement between WB and ELISA, using either the ROC-optimized cut-off or the supplier cut-off, was assessed using Cohen’s kappa correlation coefficient (22). The following scale was used to estimate the degree of agreement: 0–0.2, very weak agreement; 0.21–0.4, weak agreement; 0.41–0.6, moderate agreement; 0.61–0.8, strong agreement; and 0.81–1, almost perfect agreement (23). All statistical analyses and calculations were performed using Prism 10.6.1 (GraphPad Software, Boston, MA).

## RESULTS

### Description of the cohort

Demographic data, underlying conditions, CPA radiological subtype, and mycological data, including ELISA results, are presented in Table S1. All sera from Group 1 tested positive using the *Aspergillus* ELISA test. *Aspergillus fumigatus* (*Af*) was identified as the causative agent of CPA in 27 of 36 patients (75%). Among the remaining patients, 6 (16.6%) never had positive cultures from respiratory samples, 1 (3%) was lost to follow-up, and 1 each had *Aspergillus ochraceus* and *Aspergillus calidoustus* isolated in culture. Bronchial colonization (Group 2) and *Aspergillus* contamination (Group 3) were exclusively due to *A. fumigatus*. With a cut-off at 10 AU/mL, 12 (26.1%) and 7 (23.3%) of these patients had a positive ELISA test, respectively. Fifty-nine patients (83%) from the culture-negative patients (Group 4) and all the healthy individuals (Group 5) had a negative ELISA test.

### Optimization of the ELISA cut-off

The performance of the assay using the supplier’s proposed cut-offs of 5 and 10 AU/mL are presented in Table 1, and showed a sensitivity of 100% (95% CI [90.4%–100%]) and a specificity of 71.1% (95% CI [63.4%–77.7%]) and 83.5% (95% CI [76.8%–88.6%]), respectively.

In the control group (gathering all non-CPA patients), false-positive results were detected in 44 (29%) and 25 patients (16.5%) using the 5 and 10 AU/mL thresholds, respectively (Table S1).

**TABLE 1:** Sensitivity, specificity, negative and positive predictive values, and likelihood ratios of the ELISA *Aspergillus* IgG Platelia technique for the diagnosis of chronic pulmonary aspergillosis^*a*^^,^^*b*^^,^^*c*^

Cut-off value used (AU/mL)	Sensitivity% (95% CI)	Specificity% (95% CI)	PPV% (95% CI)	NPV% (95% CI)	LR+	LR−
≥ 5	100 (90.4–100)	71 (63.4–77.7)	0.1 (0.08–0.13)	100 (99.99–100)	3.45	0.0
≥ 10	100 (90.4–100)	83.5 (76.8–88.6)	0.19 (0.15–0.22)	100 (99.99–100)	6.1	0.0
≥ 25	100 (90.4–100)	92.8 (87.5–95.9)	0.43 (0.33–0.53)	100 (99.99–100)	13.9	0.0

^*a*^ Five and 10 AU/mL are the cut-off values recommended by the manufacturer; 25 AU/mL is the cut-off value deduced from a ROC analysis. The CPA prevalence used for the calculation of PPV and NPV was 0.000308 according to Maitre et al. (2).

^*b*^ CI, confidence interval; LR, likelihood ratio; NPV, negative predictive value; PPV, positive predictive value.

^*c*^ Thirty-six (Group 1, CPA patients) and 152 sera (control group) were used for the calculation.

**Fig 1:**
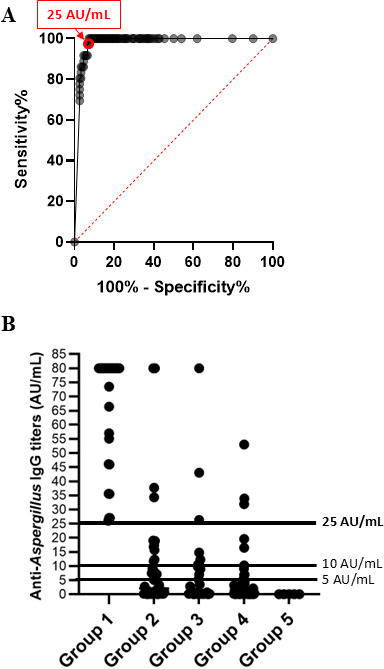
Optimization of the *Aspergillus* IgG Platelia method for diagnosing CPA patients. (**A**) ROC curve analysis based on the comparison of serum values from 36 CPA patients and 152 non-CPA patients, (**B**) Distribution of anti-*Aspergillus* IgG values obtained by ELISA across the five groups. CPA: chronic pulmonary aspergillosis.

The ROC curve analysis returned an AUC of 0.978 (95% CI [0.959–0.997]) with an optimal cut-off of 22.8 AU/mL for the diagnosis of CPA (Fig. 1A). For this value, the Youden index is maximal at 0.928 (Table S2), with a sensitivity of 100% (95% CI [90.4%–100%]) and an increased specificity of 92.8% (95% CI [87.5%–95.9%]). PPV, NPV, LR+, and LR− were at 0.43% (95% CI [0.33%–0.53%]), 100% (95% CI [99.99%–100%]), 13.9, and 0, respectively (Table 1). For practical reasons, we chose a threshold of positivity 25 AU/mL, a value that returns similar performance indexes. Using the 25 AU/mL cut-off value, 11 patients (7%) from non-CPA groups had a positive ELISA test (Fig. 1B; Table S1). Five, three, and three were in Groups 2, 3, and 4, respectively. None of these patients developed CPA or ABPA after a 6-month follow-up. In six of these 11 patients (54.5%), a concomitant viral, bacterial, or mycobacterial pulmonary infection was identified during clinical management. In two additional patients, an ongoing exacerbation was noted with no causative factor identified. Thanks to a sufficient residue of serum from eight of these patients, a second method of *Aspergillus* serology (IEP and/or WB) can be applied. Accordingly, four and six sera were also positive by IEP and WB, respectively. Four sera with high anti-*Aspergillus* IgG titers by ELISA (>80 AU/mL) were positive by both IEP and WB. Details for these patients are presented in Table S3.

### Assessment of the WB performance for the diagnosis of CPA

In the second part of this study, we aimed to evaluate the clinical performance of a commercial Western blot assay for the diagnosis of CPA. Demographic data, underlying conditions, CPA radiological subtype, and mycological data, including ELISA and WB results of this subgroup, are presented in Table S4. The sensitivity of WB for the diagnosis of CPA reached 87% (95% CI [67.9%–95.5%]). Specificity varied substantially according to the control group, ranging from 41.4% (95% CI: 25.5%–59.3%) to a maximum of 60.7% (95% CI: 42.4%–76.4%) (Table 2). Consequently, the LR+ and LR− also showed marked variability, from 1.48 to 2.21 and from 0.21 to 0.32, respectively.

**TABLE 2:** Sensitivity, specificity, negative and positive predictive values, and likelihood ratios of the LDBio *Aspergillus* Western Blot assay according to the group of patients^*a*^^,^^*b*^^,^^*c*^

Comparisons	Sensitivity% (95% CI)	Specificity% (95% CI)	PPV% (95% CI)	NPV% (95% CI)	LR+	LR−
Group 1 vs Groups 2, 3, 4, 5	87 (67.9–95.5)	50.9 (38.3–63.4)	0.05 (0.03–0.08)	99.9 (99.97–99.9)	1.77	0.25
Group 1 vs Groups 3, 4, 5		60.7 (42.4–76.4)	0.07 (0.04–0.12)	99.9 (99.9–99.9)	2.21	0.21
Group 1 vs Group 2		41.4 (25.5–59.3)	0.05 (0.03–0.07)	99.990 (99.96–99.99)	1.48	0.32
Group 1 vs Groups 2, 3, 4		46.1 (33.3–59.5)	0.05 (0.03–0.07)	99.991 (99.97–99.99)	1.66	0.23

^*a*^ Group 1 (*n* = 23): Patients with confirmed chronic pulmonary aspergillosis (CPA) according to ESCMID/ERS criteria, Group 2 (*n* = 29): Patients with Aspergillus bronchial colonization (repeated positive respiratory cultures), Group 3 (*n* = 12): Patients with suspected airway contamination (*Aspergillus* isolated from a single respiratory sample), Group 4 (*n* = 11): Patients suspected of CPA or ABPA, with negative mycological cultures and exclusion of aspergillosis after 6 months of follow-up, Group 5 (*n* = 5): Healthy controls (pregnant women).

^*b*^ The CPA prevalence used for the calculation of PPV and NPV was 0.000308 according to Maitre et al. (2).

^*c*^ CI, confidence interval; LR, likelihood ratio; NPV, negative predictive value; PPV, positive predictive value.

When Groups 2, 3, 4, and 5 were combined into a single, non-CPA, control group, specificity was at 50.9% (95% CI [38.3%–63.4%]), with LR+ and LR− at 1.77 and 0.25, respectively. Restricting the control population to Groups 3, 4, and 5 improved performances, yielding a specificity of 60.7% (95% CI [42.4%–76.4%]), and a LR+ and LR− at 2.21 and 0.21, respectively. Conversely, when Group 2 alone was used as the control group, specificity decreased to 41.4% (95% CI [25.5%–59.3%]), with a LR+ and LR− at 1.48 and 0.32, respectively. Similar results were observed when Groups 2, 3, and 4 were combined, with a specificity of 46.2% (95% CI [33.3%–59.6%]), and LR+ and LR− at 1.66 and 0.23, respectively.

The kappa correlation coefficient between WB and ELISA decreased from 0.41 (95% CI [0.22–0.61]) to 0.35 (95% CI [0.17–0.54]) when using cut-offs at 10 and 25 AU/mL, respectively.

## DISCUSSION

Measurement of *Aspergillus*-specific IgG is a cornerstone of the diagnostic criteria for CPA (8). Indeed, the clinical and radiological manifestations of CPA are often nonspecific and, in many cases, overlap with features of the underlying pulmonary condition predisposing to CPA, which can complicate timely recognition and diagnosis. Moreover, a positive *Aspergillus* culture from pulmonary samples does not necessarily reflect an active infection but may instead indicate colonization or contamination. Consequently, *Aspergillus* serology must be as specific as possible to confirm the diagnosis reliably. The primary objective of this work was to assess two serological strategies for accurate CPA diagnosis.

First, we sought to improve the specificity of our ELISA kit for diagnosing CPA by optimizing its cut-off value. Notably, ROC curve analysis suggested that increasing the cut-off to 25 AU/mL did not appear to compromise sensitivity in our data set, while specificity improved from 83.5% to 92.8%, thereby supporting the main objective of improving assay specificity. This improvement was associated with a substantial increase in the positive likelihood ratio and positive predictive value, even in the context of an extremely low disease prevalence. These findings suggest that an ELISA cut-off at 25 AU/mL may enhance the clinical utility of the assay. Nevertheless, some results were still considered false-positive results, because of our group classification. In some cases, mostly from the colonized patients’ group, these results, considered as false positives, were associated with a positive IEP. This may suggest that undiagnosed CPA was overlooked, especially since these patients presented predisposing factors for the development of CPA. This new threshold may therefore improve the detection of cases that were previously missed. This encourages close monitoring of these patients through repeated serological testing, in parallel with mycological culture of respiratory samples. Moreover, in eight patients, either a concomitant pulmonary infection with an identified non-fungal pathogen or an exacerbation due to unknown cause was observed, potentially reflecting a strong inflammatory response leading to nonspecific immune cross-reactivity with anti-*Aspergillus* IgG assays. Therefore, anti-*Aspergillus* serology in patients at risk of CPA who are being treated for a non-*Aspergillus*–related exacerbation, should be interpreted with caution. Monitoring the kinetics of anti-*Aspergillus* IgG levels after the exacerbation episode may help improve result interpretation.

We then systematically assessed the diagnostic performance of a commercial WB assay. Our study revealed a sensitivity of 87%. Whether this nonoptimal sensitivity could be due to non-*fumigatus Aspergillus* causative species should be further evaluated. In contrast, we found a low specificity, between 41.4% and 60.7%, depending on the control group. Notably, our findings suggest WB has a limited ability to distinguish between CPA and *Aspergillus*-associated conditions, including *Aspergillus* airway colonization. Consequently, the agreement between WB and ELISA, estimated with the Kappa coefficient, showed a poor concordance between the two methods, regardless of the ELISA cut-off. To explain this discrepancy, one can hypothesize that antigens used in the WB, which come from *A. fumigatus* grinding, enable the detection of anti-*Aspergillus* IgG that are not detected with the ELISA method, which only detects IgG targeting some recombinant *A. fumigatus* proteins. Whether these proteins are only or predominantly expressed during CPA and not during colonization requires complementary studies. The specificity of this WB kit varies considerably in the literature, depending on the studies and control groups they used. As an example, using healthy blood donors, specificity was calculated at 93.9% (95% CI [89.7%–96.7%]) (18). However, in another study, considering patients with suspected recurrent pulmonary tuberculosis as a control group, the specificity was at 73.5% (95% CI [61.4 to 83.5]) (24). Finally, using a large cohort of patients without any history of *Aspergillus* disease, a recent study reported a specificity of 51%, close to that observed in our study (19).

Diagnostic test performance is often evaluated using confirmed cases and healthy controls. A strength of our study is the inclusion of control groups that better reflect routine clinical practice, particularly patients with chronic respiratory diseases, clinical contexts in which anti-*Aspergillus* IgG testing is commonly performed when evaluating suspected CPA. A group of individuals without pulmonary disease or risk factors (“healthy donors”) was also included for comparison but with a smaller patient cohort. This design provides a more realistic estimate of assay specificity by including patients with predisposing factors for CPA rather than relying solely on healthy individuals.

There were several limitations in our study, the first being the quite low number of sera analyzed. This is because we wanted to analyze sera from patients before any initiation of antifungal treatment that may have introduced a bias, notably regarding the optimized cut-off value. *Aspergillus* diseases encompass multiple clinical forms that are frequently difficult to distinguish and sometimes switch from one to another. Notably, clinical signs and radiological symptoms of ABPA and CPA can overlap, and it would be useful to test whether the optimized cut-off value enables the distinction of these two clinical forms of *Aspergillus* disease.

The diagnosis of chronic pulmonary aspergillosis relies on a combination of criteria, among which a high level of *Aspergillus*-specific IgG is crucial. Our study showed that increasing the threshold value of an ELISA test can improve its specificity while maintaining excellent sensitivity. On the other hand, we demonstrated the limited value of a Western blot test in this indication, due to its relatively low specificity.

## SUPPLEMENTAL MATERIAL

Supplemental tables jcm.00182-26-s0001.docx Tables S1 to S4.

ASM does not own the copyrights to Supplemental Material that may be linked to, or accessed through, an article. The authors have granted ASM a non-exclusive, world-wide license to publish the Supplemental Material files. Please contact the corresponding author directly for reuse.
