# Supplementary material for: Aspergillus serology for chronic pulmonary aspergillosis diagnosis: optimization of an enzyme-linked immunosorbent assay kit and assessment of a Western blot kit performance
Source: J Clin Microbiol. 2026 May 28;64(7):e00182-26. doi: 10.1128/jcm.00182-26 (PMC13344010; doi:10.1128/jcm.00182-26)
Supplement: Supplemental tables — Tables S1 to S4. [file jcm.00182-26-s0001.docx]

**Supplementary Table 1**: Clinical characteristics of patients selected for the study of optimization of ELISA. CPA: chronic pulmonary aspergillosis, CCPA: chronic cavitary pulmonary aspergillosis, CFPA: chronic fibrosing pulmonary aspergillosis, CNPA: chronic necrotizing pulmonary aspergillosis, CPD: chronic pulmonary disease, COPD: chronic obstructive pulmonary disease, TB: tuberculosis, NTM: nontuberculous mycobacteria, Af: Aspergillus fumigatus, NS: not specified, SD: standard deviation. *Titre values reported as >80 UA/mL, which were outside the linear range of the assay, were assigned a value of 80 for the calculation of the mean and standard deviation.

|  | **Group 1** | **Group 2** | **Group 3** | **Group 4** | **Group 5** | **Total** |
| --- | --- | --- | --- | --- | --- | --- |
| **Patients** | CPA | *Af* bronchial colonization | *Af* bronchial contamination | Negative | Healthy donor |  |
| **N (patients and sera)** | 36 | 46 | 30 | 71 | 5 | 188 |
| **Sex ratio (M/F)** | 27/9 | 25/21 | 14/16 | 41/31 | 0/5 | 107/81 |
| **Mean age (min;max) in years** | 57 (20;81) | 42 (8;87) | 60 (5;82) | 60 (1;89) | 31 (29;38) | 54 (1;89) |
| **CPA types** |  | | | | | |
| CCPA | 13 |  |  |  |  | 13 |
| CFPA | 1 |  |  |  |  | 1 |
| CNPA | 2 |  |  |  |  | 2 |
| Aspergilloma | 6 |  |  |  |  | 6 |
| Semi-invasive | 3 |  |  |  |  | 3 |
| NS | 12 |  |  |  |  | 12 |
| **Underlying CPD** |  | | | | | |
| COPD | 6 | 9 | 7 | 15 |  | 37 |
| Emphysema | 4 | 1 | 0 | 2 |  | 7 |
| Bronchiectasis | 2 | 0 | 0 | 0 |  | 2 |
| Lung cancer | 3 | 2 | 1 | 7 |  | 13 |
| Lung TB | 1 | 1 | 2 | 1 |  | 5 |
| Lung TB scarring | 14 | 0 | 1 | 6 |  | 21 |
| NTM lung infection | 4 | 2 | 1 | 3 |  | 10 |
| Bronchial dilatation | 2 | 8 | 4 | 9 |  | 23 |
| Lung sarcoidosis | 1 | 1 | 0 | 0 |  | 2 |
| Lung fibrosis | 6 | 0 | 1 | 0 |  | 7 |
| Cystic fibrosis | 0 | 11 | 0 | 3 |  | 14 |
| Primary ciliary dyskinesia | 0 | 10 | 2 | 0 |  | 12 |
| Asthma | 1 | 6 | 7 | 16 |  | 30 |
| NS | 0 | 0 | 1 | 0 |  | 1 |
| **N of patients with *Af* in culture of at least one pulmonary sample (%)** | 27 (75) | 46 (100) | 30 (100) | 0 (0) | 0 (0) | 103 (55) |
| **Mean *Aspergillus* IgG titer (AU/mL) +/- SD*** | 69.3 +/- 18.6 | 11 +/- 20.3 | 8 +/- 16.5 | 3.3 +/- 8.7 | 0 +/- 0 | 18.5 +/- 29.3 |
| Sera with *Aspergillus* IgG titer < 5 AU/mL, N (%) | 0 (0) | 25 (54.3) | 19 (63.3) | 59 (83) | 5 (100) | 108 (57.4) |
| Sera with *Aspergillus* IgG titer ≥ 5 AU/mL and < 10 AU/mL, N (%) | 0 (0) | 9 (19.5) | 4 (13.3) | 6 (8.4) | 0 (0) | 19 (10) |
| Sera with *Aspergillus* IgG titer ≥ 10 AU/mL, N (%) | 36 (100) | 12 (26.1) | 7 (23.3) | 6 (8.4) | 0 (0) | 61 (32) |
| Sera with *Aspergillus* IgG titer ≥ 25 AU/mL, N (%) | 36 (100) | 5 (10.8) | 3 (10) | 3 (4.2) | 0 (0) | 47 (25) |

**Supplementary Table 2:** Youden’s J statistical analysis from the ROC curve. Sensitivity, specificity, and Youden index are shown for each threshold.

| **Cut-off** | **Sensitivity** | **Specificity** | **J** |
| --- | --- | --- | --- |
| > 0,05000 | 1,000 | 0,099 | 0,099 |
| > 0,1500 | 1,000 | 0,204 | 0,204 |
| > 0,2500 | 1,000 | 0,382 | 0,382 |
| > 0,3500 | 1,000 | 0,461 | 0,461 |
| > 0,4500 | 1,000 | 0,500 | 0,500 |
| > 0,5500 | 1,000 | 0,546 | 0,546 |
| > 0,6500 | 1,000 | 0,572 | 0,572 |
| > 0,7500 | 1,000 | 0,579 | 0,579 |
| > 0,9500 | 1,000 | 0,592 | 0,592 |
| > 1,150 | 1,000 | 0,605 | 0,605 |
| > 1,250 | 1,000 | 0,618 | 0,618 |
| > 1,350 | 1,000 | 0,625 | 0,625 |
| > 1,450 | 1,000 | 0,632 | 0,632 |
| > 1,550 | 1,000 | 0,638 | 0,638 |
| > 1,850 | 1,000 | 0,645 | 0,645 |
| > 2,150 | 1,000 | 0,658 | 0,658 |
| > 2,450 | 1,000 | 0,665 | 0,665 |
| > 2,750 | 1,000 | 0,671 | 0,671 |
| > 3,000 | 1,000 | 0,678 | 0,678 |
| > 3,300 | 1,000 | 0,697 | 0,697 |
| > 3,700 | 1,000 | 0,704 | 0,704 |
| > 4,500 | 1,000 | 0,711 | 0,711 |
| > 5,100 | 1,000 | 0,730 | 0,730 |
| > 5,250 | 1,000 | 0,737 | 0,737 |
| > 5,450 | 1,000 | 0,743 | 0,743 |
| > 6,250 | 1,000 | 0,750 | 0,750 |
| > 6,950 | 1,000 | 0,757 | 0,757 |
| > 7,050 | 1,000 | 0,770 | 0,770 |
| > 7,107 | 1,000 | 0,776 | 0,776 |
| > 7,296 | 1,000 | 0,783 | 0,783 |
| > 7,489 | 1,000 | 0,790 | 0,790 |
| > 7,676 | 1,000 | 0,796 | 0,796 |
| > 8,176 | 1,000 | 0,803 | 0,803 |
| > 8,700 | 1,000 | 0,809 | 0,809 |
| > 8,950 | 1,000 | 0,816 | 0,816 |
| > 9,050 | 1,000 | 0,822 | 0,822 |
| > 9,400 | 1,000 | 0,829 | 0,829 |
| > 9,950 | 1,000 | 0,836 | 0,836 |
| > 10,32 | 1,000 | 0,842 | 0,842 |
| > 10,69 | 1,000 | 0,849 | 0,849 |
| > 11,34 | 1,000 | 0,855 | 0,855 |
| > 12,00 | 1,000 | 0,862 | 0,862 |
| > 12,29 | 1,000 | 0,868 | 0,868 |
| > 13,53 | 1,000 | 0,875 | 0,875 |
| > 15,23 | 1,000 | 0,882 | 0,882 |
| > 16,10 | 1,000 | 0,888 | 0,888 |
| > 16,62 | 1,000 | 0,895 | 0,895 |
| > 17,04 | 1,000 | 0,901 | 0,901 |
| > 18,11 | 1,000 | 0,908 | 0,908 |
| > 18,93 | 1,000 | 0,915 | 0,915 |
| > 19,30 | 1,000 | 0,921 | 0,921 |
| > 22,80 | 1,000 | 0,928 | 0,928 |
| > 26,15 | 0,972 | 0,928 | 0,900 |
| > 26,65 | 0,972 | 0,934 | 0,906 |
| > 29,45 | 0,917 | 0,934 | 0,851 |
| > 32,90 | 0,917 | 0,941 | 0,858 |
| > 34,10 | 0,917 | 0,947 | 0,864 |
| > 34,90 | 0,917 | 0,954 | 0,871 |
| > 35,56 | 0,889 | 0,954 | 0,843 |
| > 36,66 | 0,861 | 0,954 | 0,815 |
| > 40,35 | 0,861 | 0,961 | 0,822 |
| > 44,45 | 0,861 | 0,967 | 0,828 |
| > 45,99 | 0,833 | 0,967 | 0,800 |
| > 49,54 | 0,806 | 0,967 | 0,773 |
| > 54,05 | 0,806 | 0,974 | 0,779 |
| > 56,05 | 0,778 | 0,974 | 0,752 |
| > 61,69 | 0,750 | 0,974 | 0,724 |
| > 69,94 | 0,722 | 0,974 | 0,696 |
| > 76,75 | 0,694 | 0,974 | 0,668 |

**Supplementary Table 3:** Clinical and serological characteristics of the 11 false-positive patients with the ELISA technique and the adapted cut-off. CPA: chronic pulmonary aspergillosis, CCPA: chronic cavitary pulmonary aspergillosis, CFPA: chronic fibrosing pulmonary aspergillosis, CNPA: chronic necrotizing pulmonary aspergillosis, *CPD:* chronic pulmonary disease*,* COPD: chronic obstructive pulmonary disease*,* TB: tuberculosis*,* NTM: nontuberculous mycobacteria*,* *Af*: *Aspergillus fumigatus,* NS: not specified, SD: standard deviation. *Titre values reported as >80 AU/mL, which were outside the linear range of the assay, were assigned a value of 80 for the calculation of the mean and standard deviation.

| **Group** | **Titre* (UA/mL)** | **N of PL in IEP (interpretation)** | **WB** | **Underlying CPD and context** | **Outcome at 6 months** |
| --- | --- | --- | --- | --- | --- |
| 3 | 26.3 | NP | NP | COPD, *Pseudomonas aeruginosa* lung infection | No progression to CPA/ABPA |
| 4 | 31.9 | 0 (N) | + | COPD, asthma, *Haemophilus influenzae* lung infection | No progression to CPA/ABPA |
| 4 | 33.9 | NP | NP | Emphysema and NTM lung infection | No progression to CPA/ABPA |
| 2 | 34.3 | 1 (N) | - | COPD, asthma, exacerbation without apparent cause | No progression to CPA/ABPA |
| 2 | 37.7 | 1 (N) | + | Emphysema | No progression to CPA/ABPA |
| 3 | 43 | 0 (N) | - | COPD, respiratory syncytial virus lung infection | No progression to CPA/ABPA |
| 4 | 53 | NP | NP | Lung cancer | No progression to CPA/ABPA |
| 2 | >80 | 2 (P) | + | COPD, asthma, exacerbation without apparent cause | No progression to CPA/ABPA |
| 3 | >80 | 2 (P) | + | 0, severe pneumonia caused by SARS-CoV-2 | No progression to CPA/ABPA |
| 2 | >80 | 4 (P) | + | COPD | No progression to CPA/ABPA |
| 2 | >80 | 5 (P) | + | Asthma, bronchial dilatation, *Pseudomonas aeruginosa* lung infection | No progression to CPA/ABPA |

**Supplementary Table 4**: Clinical characteristics of patients selected for the study of Western Blot performances. CCPA: chronic cavitary pulmonary aspergillosis, CFPA: chronic fibrosing pulmonary aspergillosis, CNPA: chronic necrotizing pulmonary aspergillosis, NS: not specified, SD: standard deviation. *Titre values reported as >80 AU/mL, which were outside the linear range of the assay, were assigned a value of 80 for the calculation of the mean and standard deviation. ** For the remaining serum samples, immunoelectrophoresis was not performed due to insufficient serum volume or unavailable serum for retrospective analysis. $ The negative Western blot results obtained in the group 1 were observed in serum samples from three patients in whom Aspergillus fumigatus was the etiologic species.

|  | **Group 1** | **Group 2** | **Group 3** | **Group 4** | **Group 5** | **Total** |
| --- | --- | --- | --- | --- | --- | --- |
| **Patients** | CPA | *Af* bronchial colonization | *Af* bronchial contamination | Negative | Healthy donor |  |
| **N (patients and sera)** | 23 | 29 | 12 | 11 | 5 | 80 |
| **Sex ratio (M/F)** | 18/5 | 16/13 | 6/6 | 9/2 | 0/5 | 49/31 |
| **Mean age (min;max) in years** | 54 (20;76) | 29 (8;77) | 58 (5;79) | 49 (1;74) | 31 (29;38) | 42 (1;76) |
| **CPA types** |  |  |  |  |  |  |
| CCPA | 7 |  |  |  |  | 7 |
| CFPA | 1 |  |  |  |  | 1 |
| CNPA | 2 |  |  |  |  | 2 |
| Aspergilloma | 4 |  |  |  |  | 4 |
| Semi-invasive | 3 |  |  |  |  | 3 |
| NS | 7 |  |  |  |  | 7 |
| **Underlying CPD** |  |  |  |  |  |  |
| COPD | 3 | 4 | 2 | 5 |  | 14 |
| Emphysema | 3 | 0 | 0 | 0 |  | 3 |
| Bronchiectasis | 2 | 0 | 0 | 0 |  | 2 |
| Lung cancer | 3 | 0 | 0 | 0 |  | 3 |
| Lung TB | 1 | 0 | 1 | 0 |  | 2 |
| Lung TB scarring | 10 | 0 | 0 | 2 |  | 12 |
| NTM lung infection | 3 | 0 | 1 | 0 |  | 4 |
| Bronchial dilatation | 1 | 1 | 0 | 0 |  | 2 |
| Lung sarcoidosis | 1 | 0 | 0 | 0 |  | 1 |
| Lung fibrosis | 1 | 0 | 0 | 0 |  | 1 |
| Cystic fibrosis | 0 | 3 | 0 | 3 |  | 6 |
| Primary ciliary dyskinesia | 0 | 5 | 2 | 0 |  | 7 |
| Asthma | 0 | 2 | 0 | 3 |  | 5 |
| NS | 0 | 0 | 0 | 0 |  | 0 |
| **N of patients with *Af* in culture of at least one pulmonary sample (%)** | 17 (74) | 29 (100) | 12 (100) | 0 (0) | 0 (0) | 58 (72.5) |
| **Mean *Aspergillus* IgG titer (AU/mL) +/- SD*** | 70.3 +/6 18.4 | 15.7 +/- (24.2) | 16.7 +/- 22.8 | 8.7 +/- 8.8 | 0.0 +/- 0.0 | 29.6 +/- 32.8 |
| Sera with *Aspergillus* IgG titer < 5 AU/mL, N (%) | 0 (0) | 12 (41.4) | 3 (25) | 3 (27.3) | 5 (100) | 23 (28.7) |
| Sera with *Aspergillus* IgG titer ≥ 5 AU/mL and < 10 AU/mL, N (%) | 0 (0) | 6 (20.7) | 3 (25) | 5 (45.5) | 0 (0) | 14 (17.5) |
| Sera with *Aspergillus* IgG titer ≥ 10 AU/mL, N (%) | 23 (100) | 11 (38) | 6 (50) | 3 (27.3) | 0 (0) | 43 (59.7) |
| Sera with *Aspergillus* IgG titer ≥ 25 AU/mL, N (%) | 23 (100) | 5 (17.2) | 2 (16.6) | 1 (9) | 0 (0) | 31 (38.7) |
| Western blot results (negative/positive) | 3^$^/23 | 12/17 | 5/7 | 7/4 | 5/0 | 32/48 |
